# Molecular Mechanisms and Targeted Therapies of PTPN2 in Metabolic Diseases: A Review

**DOI:** 10.3390/biom16071016

**Published:** 2026-07-12

**Authors:** Yue Yuan, Jing Xie, Mo Wang, Yishan Li, Xinxin Zhang, Zunjie Bo, Lei Sun, Ajing Xu

**Affiliations:** 1 Department of Clinical Pharmacy, Xinhua Hospital Affiliated to Shanghai Jiao Tong University School of Medicine, Shanghai 200092, China; yuanyue319@sjtu.edu.cn (Y.Y.); kilence0421@sjtu.edu.cn (J.X.); 7220010081@shsmu.edu.cn (M.W.); lys1527571806@163.com (Y.L.); 242322236@st.usst.edu.cn (X.Z.); b2744880422@163.com (Z.B.); 2 School of Health Science and Engineering, University of Shanghai for Science and Technology, Shanghai 200093, China; 3 Shanghai Frontiers Science Center of Drug Target Identification and Delivery, School of Pharmaceutical Sciences, Shanghai Jiao Tong University, Shanghai 200240, China

**Keywords:** PTPN2, metabolic disorders, type 2 diabetes, molecular mechanisms, therapeutic targets

## Abstract

Metabolic disorders encompass a spectrum of pathologies driven by the dysregulation of systemic metabolic homeostasis. Their escalating global prevalence has positioned these conditions at the forefront of contemporary biomedical research. Protein tyrosine phosphatase non-receptor type 2 (PTPN2) regulates cellular tyrosine phosphorylation and links metabolic perturbations to altered signal transduction. Functionally, PTPN2 modulates immune responses, cellular proliferation, and metabolic signaling pathways. Consequently, it influences immunological tolerance, glucose and lipid metabolism, and insulin sensitivity in a context-dependent manner. To facilitate the identification of novel therapeutic targets, this review systematically delineates the molecular mechanisms underlying PTPN2 function across diverse metabolic pathologies. Specifically, we examine its involvement in type 1 diabetes, type 2 diabetes, diabetic complications, and metabolic dysfunction-associated steatohepatitis. Furthermore, we highlight recent advances in PTPN2-targeted interventions, with a particular emphasis on type 2 diabetes research, while critically evaluating existing clinical challenges and future translational prospects. Ultimately, this synthesis provides an integrated perspective for the development of precision medicine strategies in the management of metabolic diseases.

## 1. Introduction

### 1.1. Overview of the Protein Tyrosine Phosphatase (PTP) Family

Protein Tyrosine Phosphatases (PTPs) constitute a specialized superfamily of enzymes that function in concert with protein tyrosine kinases (PTKs) to govern the reversible phosphorylation of tyrosine residues. These enzymes regulate intracellular signaling networks and participate in cellular processes such as growth, proliferation, differentiation, migration, adhesion, and apoptosis [1]. Notably, the dysregulation of PTP activity is inextricably linked to the pathogenesis of diverse pathologies, such as diabetes, cancer, autoimmune disorders, and osteoporosis [2,3,4,5]. Consequently, elucidating the functional roles of PTPs is essential for decoding the molecular underpinnings of these diseases and for the development of novel therapeutic interventions.

The protein tyrosine phosphatase (PTP) family comprises a large and structurally diverse group of enzymes. Based on the identity of their catalytic nucleophilic residues (aspartate, cysteine, or histidine) and structural topology, PTPs can be broadly classified into three major subfamilies: aspartate-based PTPs, cysteine-based PTPs, and histidine-based PTPs [6]. Among these, aspartate-based and histidine-based PTPs together comprise only nine enzymes, whereas cysteine-based PTPs represent the largest subfamily, encompassing 117 members and further subdivided into Class I, Class II, and Class III phosphatases [7].

**Figure 1:**
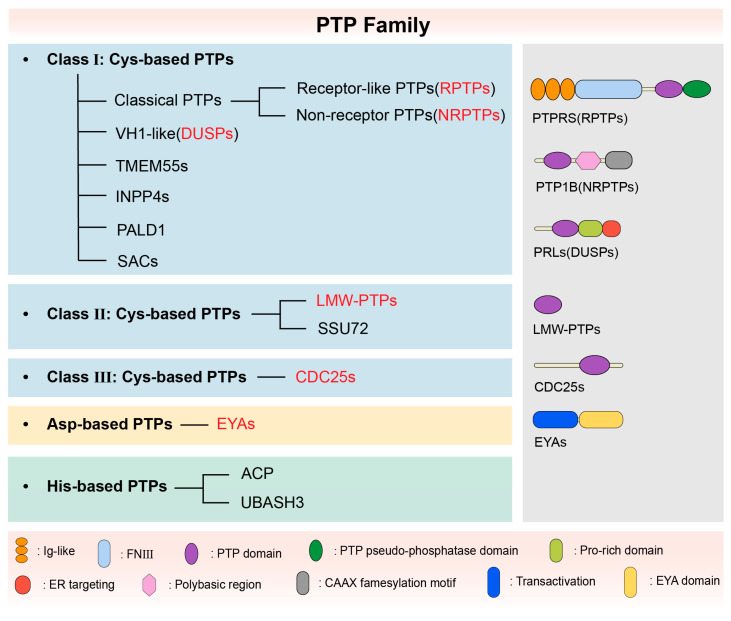
Classification and Representative Structures of the PTP Superfamily. Based on catalytic domain homology, the PTP superfamily can be classified into three major groups: aspartate-based PTPs, cysteine-based PTPs, and histidine-based PTPs. Among these, cysteine-based PTPs constitute the largest group and can be further subdivided into Class I, Class II, and Class III phosphatases. Class I PTPs are further organized into two major branches: classical tyrosine-specific phosphatases (Classical PTPs) and dual-specificity phosphatases (DUSPs). Classical PTPs selectively dephosphorylate phosphotyrosine (pTyr) residues and include both receptor-type PTPs (RPTPs), which possess transmembrane domains (e.g., PTPRS), and intracellular non-receptor-type PTPs (NRPTPs) (e.g., PTPN1, also known as PTP1B). In contrast, DUSPs are capable of dephosphorylating both phosphotyrosine (pTyr) and phosphoserine/phosphothreonine (pSer/pThr) residues and include members such as the phosphatase of regenerating liver (PRL) family [10,11,12,13,14].

Within the Class I PTP family, phosphatases can be further categorized into two major branches based on substrate specificity and structural characteristics: classical tyrosine-specific phosphatases (Classical PTPs) and dual-specificity phosphatases (DUSPs). Classical PTPs specifically catalyze the dephosphorylation of phosphotyrosine (pTyr) residues and can be further subdivided into receptor-type PTPs (RPTPs), which contain transmembrane domains, and non-receptor-type PTPs (NRPTPs), which are localized to the cytoplasm or other subcellular compartments. In contrast, DUSPs constitute a distinct branch within the Class I PTP family and are capable of dephosphorylating both phosphotyrosine (pTyr) and phosphoserine/phosphothreonine (pSer/pThr) residues [8]. In comparison, Class II and Class III PTPs are considerably smaller subfamilies. Class II PTPs comprise only low-molecular-weight protein tyrosine phosphatases (LMW-PTPs) and SSU72, whereas Class III PTPs consist exclusively of three tyrosine-/threonine-specific phosphatases: CDC25A, CDC25B, and CDC25C [9]. The classification and representative structures of the PTP superfamily are summarized in Figure 1.

### 1.2. Structure and Function of PTPN2

Among Class I non-receptor PTPs, protein tyrosine phosphatase non-receptor type 2 (PTPN2), also known as T-cell protein tyrosine phosphatase (TC-PTP), has attracted increasing attention. Functioning as a pivotal node in intracellular signaling networks, PTPN2 exerts negative regulatory control over diverse pathways essential for physiological homeostasis, including those governing immunity, cellular proliferation, and metabolism. Consequently, aberrant PTPN2 activity is inextricably linked to the etiology of autoimmune disorders, malignancies, and metabolic dysregulation [4,15,16], thereby positioning this enzyme as a compelling candidate for therapeutic intervention.

Originally identified from a human T-cell cDNA library, the PTPN2 gene is located on chromosome 18p11.21. Its coding sequence comprises 10 exons, with exons 1–7 encoding the highly conserved protein tyrosine phosphatase (PTP) catalytic domain [17].

**Figure 2:**
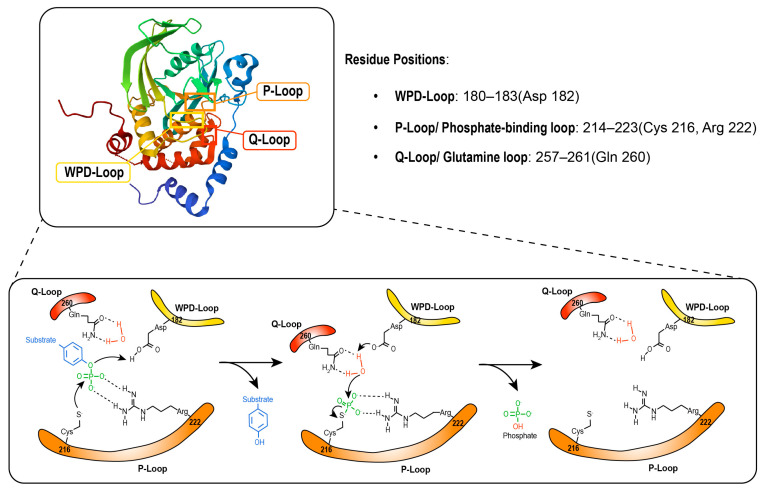
Catalytic mechanism of substrate dephosphorylation by PTPN2. The catalytic residue Cys216 initiates substrate dephosphorylation through a nucleophilic attack on the phosphate group of the phosphotyrosine substrate, whereas Asp182 facilitates release of the dephosphorylated substrate by acting as a proton donor. Subsequently, hydrolysis of the phosphocysteine intermediate by an activated water molecule regenerates the catalytic Cys216 residue and completes the catalytic cycle. The structural model shown is based on the crystal structure of the PTPN2 catalytic domain. The WPD loop, P-loop, and Q-loop are highlighted in yellow, orange, and red, respectively (PDB ID: 7F5O). Arrows indicate the direction of the catalytic reaction.

Like other classical PTPs, PTPN2 catalyzes substrate dephosphorylation through a two-step mechanism involving the formation and subsequent hydrolysis of a phosphocysteine intermediate [7,18]. Upon substrate binding, the WPD loop undergoes a conformational transition from an open to a closed state, positioning the catalytic aspartate residue (Asp182) in close proximity to the phosphorylated tyrosine of the substrate. In the first catalytic step, the thiolate group of the conserved catalytic cysteine residue (Cys216) within the P-loop performs a nucleophilic attack on the phosphorus atom of the phosphate group. Simultaneously, Asp182 acts as a general acid by donating a proton to the leaving tyrosine oxygen, thereby facilitating cleavage of the phosphorus–oxygen bond and release of the dephosphorylated substrate. This reaction results in the formation of a covalent phosphocysteine intermediate. The second, rate-limiting step involves hydrolysis of the phosphocysteine intermediate. During this process, Asp182 switches its role from a general acid to a general base, abstracting a proton from a water molecule to promote its activation. The conserved glutamine residue (Gln260) within the Q-loop precisely positions the activated water molecule for nucleophilic attack on the phosphorus–sulfur bond of the intermediate. Subsequent hydrolysis releases inorganic phosphate and restores the enzyme to its catalytically active state. Throughout the catalytic cycle, the conserved arginine residue (Arg222) in the P-loop stabilizes the negatively charged transition state and coordinates the phosphate group, thereby enhancing catalytic efficiency. Collectively, the catalytic activity of PTPN2 depends on the coordinated actions of multiple conserved structural elements, including the P-loop, WPD loop, and Q-loop, whereas mutations affecting these key residues typically result in a marked reduction in phosphatase activity. The catalytic mechanism of substrate dephosphorylation by PTPN2 is illustrated in Figure 2.

Alternative splicing of the PTPN2 transcript generates two predominant isoforms designated as TC48 (48 kDa) and TC45 (45 kDa). Although these variants share an identical N-terminal sequence spanning approximately 365 to 370 amino acids that houses the catalytic domain, they diverge significantly in their C-terminal architecture. This structural variation dictates their specific subcellular compartmentalization and functional diversity [17]. The TC48 isoform is encoded by exons 1 through 8. It is distinguished by a unique C-terminal hydrophobic domain of approximately 19 residues encoded by exon 8 which anchors the enzyme to the cytoplasmic face of the endoplasmic reticulum where it modulates metabolic pathways [19]. Conversely, TC45 is derived from exons 1 through 7 spliced to a segment of exon 9. Lacking the hydrophobic anchor, TC45 features a short hydrophilic tail encoded by exon 9. Consequently, this isoform localizes primarily to the nucleus to orchestrate processes such as immune modulation, inflammation suppression, cell differentiation, cell cycle progression, and DNA repair [20]. However, the subcellular distribution of PTPN2 isoforms is dynamically regulated rather than being fixed. Although TC45 is predominantly localized in the nucleus under basal conditions, stimulation by growth factors such as epidermal growth factor (EGF) or inflammatory cytokines can promote its nucleocytoplasmic shuttling, enabling access to cytoplasmic substrates, including JAKs, STATs, and receptor tyrosine kinases (RTKs) [21]. These observations suggest that the functional diversity of PTPN2 isoforms is shaped not only by their structural differences but also by stimulus-dependent changes in subcellular localization. The domain architecture and subcellular localization characteristics of the two PTPN2 isoforms are summarized in Table 1.

## 2. Mechanistic Role of PTPN2 in Metabolic Diseases

### 2.1. PTPN2 in Type 1 Diabetes (T1D)

Type 1 diabetes (T1D) manifests as a complex, T-cell-mediated autoimmune pathology culminating in the progressive attrition of pancreatic β-cells and a consequent decline in insulin secretory capacity. Despite extensive research, the precise etiology of T1D remains incompletely defined. Clinically, the current therapeutic landscape is strictly limited to lifelong exogenous insulin administration, underscoring the critical absence of curative or disease-modifying alternatives [22].

Converging lines of evidence implicate PTPN2 in the pathogenesis of T1D through both genetic and environmental mechanisms. From a genetic perspective, Genome-Wide Association Studies (GWASs) identified PTPN2 as a principal susceptibility locus. Subsequent investigations utilizing genetic colocalization and Mendelian randomization have suggested that DNA methylation modifications at this locus mediate genetic risk by modulating PTPN2 expression [23]. Consequently, this transcriptional dysregulation impairs the capacity of the enzyme to modulate islet immune responses and mitigate deleterious inflammation. At the environmental level, PTPN2 is involved in the regulation of antiviral immune responses, and viral infection has been recognized as an important environmental factor associated with T1D development. Experimental studies suggest that reduced PTPN2 expression may increase the susceptibility of pancreatic β-cells to virus-induced apoptosis, thereby potentially contributing to β-cell loss during disease progression [24,25].

#### 2.1.1. Genetic Control of PTPN2 in Type 1 Diabetes Pathogenesis

From a genetic perspective, PTPN2 polymorphisms, particularly specific single nucleotide polymorphisms (SNPs), have been associated with an increased risk of T1D. Most of these variants are thought to influence disease susceptibility primarily by altering PTPN2 expression rather than directly affecting protein structure or enzymatic activity. Reduced PTPN2 expression may impair immune regulation and diminish the protective effects of PTPN2 on pancreatic β-cells, thereby promoting autoimmune-mediated β-cell destruction and increasing susceptibility to T1D. Notably, empirical studies have demonstrated that the majority of T1D-associated SNPs, including rs1893217, rs2542151, and rs478582, are correlated with downregulated PTPN2 expression [26,27].

The bidirectional crosstalk between pancreatic β-cells and the immune system constitutes a pivotal determinant in the pathogenesis of T1D [28]. Accordingly, the subsequent analysis delineates the regulatory mechanisms governed by PTPN2 within both the immune compartment and the pancreatic β-cell niche.

T cell-specific deficiency of PTPN2 has been shown to enhance autoimmune responses and promote immune-mediated destruction of pancreatic β-cells. In vivo studies using non-obese diabetic (NOD) mice demonstrated that T cell-specific deletion of PTPN2 significantly increases the incidence of T1D, as well as other autoimmune disorders, including colitis and Sjögren’s syndrome [2]. Mechanistically, PTPN2 regulates T-cell homeostasis through at least two complementary pathways. First, it negatively regulates T-cell receptor (TCR) signaling, thereby establishing the activation threshold of naïve T cells and preventing excessive T-cell activation [29]. Second, PTPN2 attenuates T-cell responsiveness to inflammatory cytokines, including interferon-γ (IFN-γ) and interleukin-2 (IL-2), by dephosphorylating key signaling molecules such as JAK1/3 and STAT1/3/5, thereby limiting aberrant T-cell expansion under inflammatory conditions [30]. Loss of these regulatory mechanisms can disrupt immune tolerance, rendering T cells more prone to recognizing and attacking pancreatic β-cells, ultimately leading to progressive β-cell destruction through cytotoxic and inflammatory processes [28]. In addition, dysregulated PTPN2 signaling in other immune cell populations, including B cells and dendritic cells, may also contribute to the initiation and progression of T1D [2].

PTPN2 serves as a vital cytoprotective agent that shields pancreatic β-cells from cytokine-induced toxicity [28]. Mechanistically, PTPN2 directly attenuates the hyperactivation of the JAK/STAT signaling, thereby functioning as a critical negative regulator of interferon signaling [31]. Furthermore, research by Elvira et al. indicates that the protective efficacy of PTPN2 extends beyond signal transduction. It mitigates endoplasmic reticulum (ER) stress and modulates the unfolded protein response (UPR), collectively preserving β-cell integrity against autoimmune insults [32]. Conversely, PTPN2 deficiency heightens β-cell susceptibility to pro-inflammatory cytokines, such as IFN-α and tumor necrosis factor-α (TNF-α). This sensitization precipitates deleterious outcomes concomitant with mitochondrial dysfunction [33,34]. Of critical importance is that these pathological alterations exacerbate the immune response. As demonstrated in PTPN2-deficient β-cell models, cellular stress drives the upregulation of HLA class I molecules. This process enhances β-cell immunogenicity and provokes robust autoreactive T-cell responses that accelerate the progression of T1D [35].

#### 2.1.2. Environmental Control of PTPN2 in Type 1 Diabetes Pathogenesis

**Figure 3:**
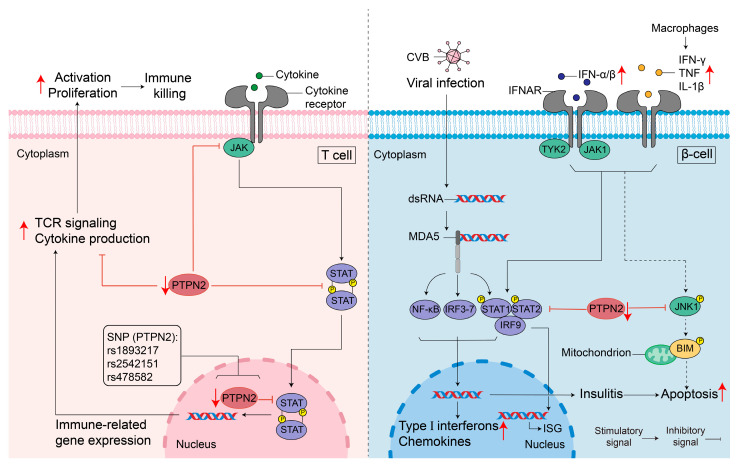
Molecular Mechanisms of PTPN2 in T Cells and β-Cells during T1D Pathogenesis. T-Cell Regulation (Left Panel): The ablation of PTPN2 in T cells amplifies STAT-mediated cytokine expression and TCR signaling responses. This dysregulation potentiates T cell activation and subsequently augments T cell-mediated cytotoxicity against β-cells. β-Cell Antiviral and Apoptotic Signaling (Right Panel): Coxsackievirus B (CVB) infection generates cytoplasmic double-stranded RNA (dsRNA). This viral RNA is sensed by the cytosolic receptor MDA5, which recruits and activates transcription factors including NF-κB, IRFs, and STATs. Consequently, this leads to the production of type I interferons and chemokines that culminate in local inflammation or pancreatitis. Mechanistically, type I interferons bind to the IFN-α/β receptor (IFNAR) and initiate signaling via TYK2 and JAK1. This cascade induces STAT activation and the transcription of interferon-stimulated genes (ISGs) to establish an antiviral state. Concurrently, exposure to type I interferons, type II interferons (IFN-γ), TNF, and IL-1β promotes JNK1 activation. Activated JNK1 triggers the phosphorylation of the pro-apoptotic protein BIM (P-BIM) and precipitates the mitochondrial apoptotic pathway. PTPN2 acts as a protective checkpoint by negatively regulating STAT signaling and inhibiting JNK1-mediated phosphorylation of BIM, thereby mitigating interferon-induced β-cell death. Key: Kinases and phosphatases are depicted as green ovals, with PTPN2 distinctively highlighted in red. Transcription factors are represented by purple ovals, whereas the yellow oval represents BIM. Solid lines denote direct interactions, whereas dashed lines indicate indirect regulatory effects. The meanings of the arrows and inhibitory lines are indicated in the figure.

Research by Beeck et al. identifies enteroviruses, particularly the Coxsackievirus B (CVB) group, as pivotal environmental precipitants of T1D. Following infection, host cells initiate signaling cascades that activate interferon-stimulated genes (ISGs) to establish an antiviral state. PTPN2 functions as a critical modulator of this antiviral response in both β-cells and the immune system [24], exerting a dichotomous influence based on its expression levels. Physiologically, PTPN2 serves to prevent tissue damage resulting from immune hyperactivation. Studies indicate that the suppression of PTPN2 in β-cells amplifies STAT signaling and exacerbates pancreatic inflammation. Furthermore, this deficiency sensitizes cells to apoptosis induced by IFN-α, IFN-β, and IFN-γ via the mitochondrial pathway. Conversely, the aberrant overexpression of PTPN2 significantly compromises the efficacy of viral clearance. This dysregulation potentially predisposes the host to chronic viral persistence or facilitates augmented viral replication [36]. The molecular mechanisms by which PTPN2 regulates T-cell activation and β-cell anti-viral responses during T1D pathogenesis are illustrated in Figure 3.

### 2.2. PTPN2 in Type 2 Diabetes (T2D)

Type 2 diabetes (T2D) represents a chronic metabolic pathology characterized by the dual hallmarks of insulin resistance and the progressive functional decline of pancreatic β-cells. Among the contributing factors, visceral adiposity acts as a primary driver of disease pathogenesis [37,38,39]. Within this complex metabolic landscape, PTPN2 exerts a multifaceted regulatory influence. Specifically, the enzyme exhibits bidirectional effects that vary depending on the specific tissue context and signaling dynamics.

#### 2.2.1. PTPN2-Mediated Regulation of Insulin Resistance

Insulin resistance constitutes a prevalent metabolic perturbation characterized by the attenuated sensitivity of insulin-responsive tissues to insulin. This condition compromises the capacity of target tissues to effectively execute glucose uptake, suppress endogenous glucose production and lipolysis, and stimulate glycogen synthesis, notwithstanding the presence of elevated plasma insulin concentrations [40,41]. PTPN2 modulates this pathological state through multifaceted mechanisms. Its regulatory influence encompasses tissue-specific actions within the hypothalamus, liver, adipose tissue, and skeletal muscle. Furthermore, PTPN2 intervenes systematically in chronic inflammatory pathways, which constitute a fundamental pathological cornerstone of T2D.

In the hypothalamus, PTPN2 has been identified as an important regulator of central insulin resistance [42]. Agouti-related peptide (AgRP) neurons function as essential regulators of appetite, energy homeostasis, and glucose metabolism [43]. Under physiological conditions, postprandial insulin surges suppress the activity of these neurons by activating insulin receptor (IR) signaling. This process is integral to satiety induction and the optimization of systemic glucose metabolism [44,45]. PTPN2 attenuates this signaling pathway through dephosphorylation of the insulin receptor (IR), thereby reducing downstream insulin signaling and promoting inappropriate activation of AgRP neurons. Research by Dodd et al. demonstrates that the specific ablation of PTPN2 within AgRP neurons ameliorates this dysfunction. This intervention reinstates IR-mediated regulation over AgRP neurons, enhances glucose uptake in brown adipose tissue (BAT), and suppresses hepatic glucose production (HGP). Collectively, these changes significantly improve systemic insulin sensitivity [42]. Furthermore, obesity induces the upregulation of PTPN2 expression in the hypothalamus. In this context, PTPN2 dephosphorylates STAT3 to attenuate leptin signaling. This molecular blockade precipitates central leptin resistance, disrupts systemic glucose homeostasis, and exacerbates insulin resistance [46].

Research by Gurzov et al. identifies hepatic PTPN2 as a regulatory link between obesity-associated oxidative stress and hepatic insulin resistance [47]. In the context of obesity, elevated hepatic levels of reactive oxygen species (ROS) promote the oxidative inactivation of PTPN2. This event instigates a pathological pattern of “selective insulin resistance.” Specifically, the canonical insulin-mediated PI3K/AKT pathway is suppressed, whereas insulin-dependent activation of the STAT5 signaling pathway is aberrantly hyperactivated. This upregulated insulin-STAT5 axis exacerbates systemic obesity by increasing IGF-1 levels, which subsequently suppresses central growth hormone secretion. Moreover, the pathway directly drives hepatic de novo lipogenesis and steatosis. Consequently, these metabolic disturbances further aggravate insulin resistance through mechanisms of lipotoxicity [47].

**Figure 4:**
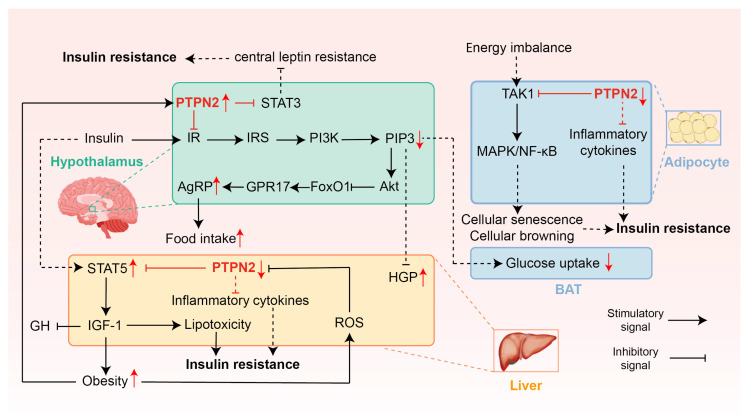
Tissue-Specific Regulatory Mechanisms of PTPN2 in Type 2 Diabetes. PTPN2 exerts distinct and occasionally divergent regulatory effects on metabolic processes within the hypothalamus (green box), liver (yellow box), and brown adipose tissue (blue box). Concurrently, the phosphatase functions as a cytoprotective agent for pancreatic β-cells. It achieves this by dephosphorylating key signaling mediators, primarily JAKs, thereby inhibiting pro-inflammatory cytokine pathways. As the mechanisms underlying β-cell inflammatory injury are discussed in detail in the T1D section, this figure focuses on the differential and shared signaling pathways regulated by PTPN2 in T1D and T2D, highlighting its role as an integrator of immune and metabolic signaling. Key: IR, insulin receptor; IRS, insulin receptor substrate; HGP, hepatic glucose production; IGF-1, insulin-like growth factor 1; GH, growth hormone. Solid lines denote direct interactions, whereas dashed lines indicate indirect effects. Red arrows indicate the upregulation or downregulation of the indicated molecules, or an increase or decrease in the corresponding biological processes.

Impaired adipose tissue browning contributes to insulin resistance and metabolic dysregulation in T2D [48]. Conversely, PTPN2 has been identified as a regulator of the adipose browning program, suggesting a potential mechanism for counteracting this defect. In the state of chronic positive energy balance characteristic of T2D, the accumulation of senescent adipocytes leads to diminished thermogenic capacity within brown adipose tissue (BAT). This decline in energy expenditure further exacerbates systemic insulin resistance. Elucidating the molecular basis of this process, Liu et al. demonstrated that PTPN2 directly interacts with and dephosphorylates transforming growth factor-activated kinase 1 (TAK1). Consequently, this interaction suppresses downstream MAPK and NF-κB signaling cascades in adipocytes. This regulatory axis effectively restores the browning phenotype and ameliorates adipose tissue insulin resistance [16]. The tissue-specific regulatory mechanisms of PTPN2 in type 2 diabetes are summarized in Figure 4.

In contrast to its regulatory roles in hepatic and adipose tissues, current evidence indicates that PTPN2 has a limited role in skeletal muscle under basal conditions. Canonically, insulin stimulates glucose uptake in myocytes through the activation of the insulin receptor tyrosine kinase and the downstream phosphoinositide 3-kinase (PI3K)/Akt signaling cascade [49,50]. However, in vivo investigations reveal that skeletal muscle-specific PTPN2-deficient mice display no discernible perturbations in glucose homeostasis or insulin sensitivity. Notably, this phenotypic resilience persists under both standard chow and high-fat diet (HFD) regimens [49]. Collectively, these findings suggest that skeletal muscle PTPN2 is not a major determinant of systemic glucose metabolism under basal conditions.

Chronic low-grade inflammation serves as a foundational etiological factor for insulin resistance, affecting key metabolic organs including adipose tissue, the liver, and skeletal muscle [51]. To investigate this interplay, Stein et al. utilized murine C2C12 myotubes to recapitulate an inflammatory milieu in vitro. Their findings demonstrated that prolonged exposure to pro-inflammatory cytokines, specifically TNF, IL-1β, and IL-6, precipitated a significant downregulation of Insulin Receptor Substrate 1 (IRS1) protein abundance. This alteration consequently compromised insulin signaling integrity [52]. Of particular significance was the observation that IL-6 specifically induced the upregulation of PTPN2 expression. Corroborating a functional link, the silencing of PTPN2 via small interfering RNA (siRNA) resulted in a marked enhancement of glucose uptake. These data suggest that inflammation-mediated upregulation of PTPN2 may contribute to impaired insulin responsiveness in skeletal muscle cells. 

#### 2.2.2. PTPN2-Mediated Preservation of β-Cell Function in T2D

Consistent with the cytoprotective mechanisms described in T1D, PTPN2 contributes to the maintenance of β-cell integrity within the chronic inflammatory milieu of T2D. Specifically, β-cells depend on adequate PTPN2 expression to withstand cytokine-induced cytotoxicity. Mechanistically, PTPN2 functions by dephosphorylating members of the JAK and STAT families. This enzymatic activity attenuates the deleterious signaling cascades triggered by pro-inflammatory factors. Consequently, this protective mechanism may help preserve insulin secretory capacity and sustain β-cell viability. Conversely, the absence or downregulation of PTPN2 significantly exacerbates the susceptibility of β-cells to inflammation-mediated destruction [31,53].

### 2.3. Mechanistic Role of PTPN2 in Diabetic Complications

A substantial proportion of patients with diabetes eventually develop cardiovascular disease and microvascular pathologies. Emerging evidence implicates PTPN2 as a pivotal regulator in the progression of these complications. A study by Keindl et al. demonstrated that polymorphisms in the IL2RA and PTPN2 genes are associated with elevated plasma levels of soluble IL-2 receptor (sIL-2R) and altered T-cell subset distributions in patients with T1D, suggesting that these immunological alterations may contribute to the development of vascular complications [54]. In addition, PTPN2 has been shown to suppress the activation of STAT1 and STAT3 in the kidney, thereby reducing the expression of pro-inflammatory and pro-fibrotic mediators, including TNF-α, IL-6, and monocyte chemoattractant protein-1 (MCP-1), as well as limiting the infiltration of CD3+ T lymphocytes and F4/80+ macrophages. These effects have been validated in both glomerular mesangial cells (MCs) and renal tubular epithelial cells (MCTs), indicating that PTPN2 may alleviate renal injury and fibrosis in diabetic nephropathy by attenuating STAT-mediated renal microinflammation [55].

Furthermore, PTPN2 functions as a critical protective agent against diabetes-associated periodontitis. Research by Zhang et al. identified PTPN2 as a direct downstream target of 25-hydroxyvitamin D_3_. Activation of this pathway effectively mitigates inflammation and attenuates bone resorption in murine models of the disease. Mechanistically, PTPN2 exerts its osteoprotective effects by targeting the colony-stimulating factor 1 receptor (CSF-1R). Through direct protein–protein interaction, PTPN2 dephosphorylates CSF-1R at the Tyr807 residue. This enzymatic action inhibits signaling cascades responsible for alveolar bone resorption [56]. Consequently, PTPN2 and CSF-1R emerge as promising therapeutic targets for the management of diabetic periodontitis and other osteolytic pathologies.

Although the clinical pipeline for therapeutics directly targeting diabetic complications remains in its infancy, a comprehensive elucidation of PTPN2 mechanisms is imperative. It is particularly critical to delineate the enzyme’s regulatory profiles and pathophysiological functions within specific tissue microenvironments. Such insights are foundational for the identification of novel druggable targets and the advancement of more efficacious therapeutic strategies.

### 2.4. PTPN2 in Broader Metabolic Pathologies

#### 2.4.1. PTPN2 Regulation in Metabolic Dysfunction-Associated Steatohepatitis (MASH)

Metabolic dysfunction-associated steatohepatitis (MASH) constitutes the advanced stage of metabolic dysfunction-associated steatotic liver disease (MASLD) characterized by established hepatic inflammation and injury, a pathological continuum predominantly driven by obesity and type 2 diabetes (T2D) [57]. Within this pathological context, PTPN2 functions as a pivotal regulator of hepatic metabolic homeostasis and immune modulation. Consequently, the dysfunction of this enzyme precipitates the onset and accelerates the progression of MASH. It drives these outcomes by exacerbating metabolic dysregulation and amplifying hepatic inflammation [58].

The molecular pathophysiology of PTPN2 in MASH is intrinsically linked to the dysregulation of oxidative stress responses and downstream signaling cascades. During the progression of MASH, markedly exacerbated hepatic oxidative stress drives the oxidative modification of protein tyrosine phosphatases, notably PTPN2, within hepatocytes, thereby precipitating their catalytic inactivation. Research by Grohmann et al. demonstrates that the inactivation of hepatocyte PTPN2 results in the hyperactivation of STAT1- and STAT3-mediated gene expression profiles. These transcriptional changes are critical for facilitating T cell infiltration, modulating liver repair mechanisms, and shaping immune responses. Collectively, these processes propel the progression of MASH and hepatic fibrosis [58]. Specifically, the amplification of STAT1 signaling promotes T cell recruitment and exacerbates hepatic inflammation. This cascade accelerates the transition from simple steatosis to MASH. Distinct from this process, the activation of STAT3 signaling functions as an independent driver of hepatocellular carcinoma (HCC) development, irrespective of the underlying MASH status.

#### 2.4.2. PTPN2 in Metabolic Bone Disease

Metabolic bone diseases constitute a heterogeneous class of disorders defined by the perturbation of intrinsic skeletal homeostasis. This disruption manifests as aberrations in bone mass, microstructural integrity, or mineral composition. Clinically, these structural deficits culminate in skeletal fragility, intractable pain, and a heightened susceptibility to fractures. Fundamentally, these pathologies arise from dysregulation within the bone remodeling apparatus itself [59]. Within this context, PTPN2 functions as a critical regulator governing both immune responses and bone metabolism. It achieves this by dephosphorylating specific signaling substrates. Notably, the systemic or lineage-specific ablation of PTPN2 severely compromises immune function. This loss of function concurrently precipitates the onset of metabolic bone disorders [60].

PTPN2 acts as a pivotal regulator of osteoimmunology, exerting control over skeletal metabolism by modulating macrophage, T-cell, and B-cell functions [60].

In macrophages, PTPN2 negatively regulates macrophage activation and the production of pro-inflammatory mediators through multiple signaling pathways, thereby limiting bone resorption. Mechanistic studies have shown that PTPN2 directly dephosphorylates Tyr807 of the CSF-1R and its downstream effector ERK, thereby suppressing CSF-1-induced osteoclast precursor differentiation, which represents a major mechanism underlying its anti-resorptive effects. In addition, PTPN2 can inhibit NLRP3 inflammasome assembly and IL-1β release through JNK dephosphorylation, thereby indirectly attenuating inflammation-associated bone resorption. Notably, PTPN2 also restrains macrophage responsiveness to lipopolysaccharide (LPS) and IFN-γ, helping to maintain a lower M1/M2 macrophage ratio and preventing excessive bone resorption driven by M1 macrophage polarization [56,61,62].

In T cells, PTPN2 maintains bone homeostasis by preserving Regulatory T cell (Treg) stability; specifically, it dephosphorylates STAT3 to prevent the pathogenic loss of Foxp3 [63]. Concomitantly, it fine-tunes Treg differentiation via IL-2 receptor signaling (targeting JAK1/3 and STAT5) and mitigates inflammation by suppressing T-cell receptor (TCR)-mediated Lck phosphorylation [29,64].

Furthermore, PTPN2 exerts context-dependent control over B-cell lineages, promoting maturation via IFN-γ/STAT1 inhibition while restricting aberrant proliferation through the IL-21/STAT3 axis, thereby sustaining the equilibrium of the bone marrow microenvironment [65,66].

Notably, the effects of IFN-γ on bone metabolism are highly dependent on both cellular context and signaling intensity. Within the monocyte–macrophage/osteoclast precursor lineage, IFN-γ directly suppresses RANKL-induced osteoclastogenesis by promoting TRAF6 degradation and inhibiting NFATc1 expression [67]. In contrast, under inflammatory conditions, IFN-γ can drive macrophage polarization toward the pro-inflammatory M1 phenotype, thereby enhancing the production of inflammatory mediators and indirectly promoting osteoclast formation and bone resorption [60].

The biological effects of IFN-γ are largely determined by the magnitude of downstream JAK/STAT signaling, which is tightly regulated by protein tyrosine phosphatases. PTPN2 attenuates JAK/STAT1 signaling and reduces macrophage responsiveness to IFN-γ stimulation, thereby suppressing M1 polarization and helping to maintain the dynamic balance between M1 and M2 macrophages [60]. Given that M1 macrophages represent a major source of pro-inflammatory mediators that promote inflammatory bone loss, this regulatory mechanism may contribute indirectly to the maintenance of bone homeostasis. Beyond myeloid cells, PTPN2-mediated regulation of IFN-γ signaling may also influence the bone marrow lymphoid compartment. During B-cell development, appropriately regulated IFN-γ/STAT1 signaling contributes to the maintenance of the bone marrow stromal microenvironment. By modulating the intensity of this pathway, PTPN2 participates in the transition from precursor B cells to mature B cells, thereby indirectly supporting immune homeostasis within the bone marrow niche [65]. The osteoimmunological regulatory effects of PTPN2 on different immune cell populations are summarized in Table 2.

### 2.5. Cross-Talk in PTPN2 Signaling: Immunoregulation and Tumorigenesis

PTPN2, alternatively known as T-cell protein tyrosine phosphatase (TC-PTP), functions as a ubiquitously expressed enzyme across human tissues. Beyond its established function as a master regulator of metabolic homeostasis, the phosphatase operates as a critical signaling nexus. It effectively bridges the intricate interface between immune surveillance architectures and the machinery governing cellular proliferation.

Under physiological conditions, PTPN2 functions as a negative regulator of immune homeostasis. Its primary molecular mechanism entails the dephosphorylation of critical kinases and transcription factors within the JAK/STAT signaling architecture. This enzymatic activity constrains both the amplitude and duration of pro-inflammatory cytokine signaling. Consequently, this modulation prevents the hyperactivation of immune responses and averts aberrant autoimmune aggression against host tissues [17]. This negative regulation of inflammatory signaling contributes to the maintenance of immune homeostasis and may reduce susceptibility to autoimmune diseases, including type 1 diabetes and inflammatory bowel disease [34,68]. Moreover, it provides a mechanistic basis for the complex interplay between PTPN2-mediated immune regulation and tumor progression.

In the context of tumor pathology, PTPN2 exhibits a dichotomy of function governed by high tissue specificity and microenvironmental dependence. Initially, the phosphatase acts as a tumor suppressor by inhibiting the early stages of specific malignancies. For instance, in T-cell acute lymphoblastic leukemia (T-ALL), genomic deletions or the functional inactivation of PTPN2 serve as potent drivers of tumorigenesis. Mechanistic studies have shown that PTPN2 deficiency enhances the responsiveness of leukemia cells to cytokines such as IL-7, leading to hyperactivation of the JAK/STAT signaling pathway and consequent promotion of cellular proliferation. In addition, loss of PTPN2 relieves its negative regulatory effect on the oncogenic kinase NUP214–ABL1, thereby further amplifying downstream pro-tumorigenic signaling pathways [69]. Furthermore, in HCC, the tumor-suppressive function of PTPN2 is compromised by environmental factors. Obesity-induced hepatic oxidative stress precipitates the inactivation of the enzyme. This event potentiates STAT3 signaling and subsequently promotes the progression of HCC [58].

Conversely, within the microenvironment of the majority of solid tumors, PTPN2 undergoes a functional inversion. It primarily fosters tumor progression by orchestrating immune evasion [7]. Recent CRISPR-based genome-wide screening studies have substantiated that tumor cells exploit PTPN2 to dephosphorylate JAK1 and STAT1 downstream of the IFN-γ receptor. This enzymatic activity significantly attenuates cellular sensitivity to IFN-γ secreted by cytotoxic T cells [15]. By abrogating the interferon signaling cascade, PTPN2 empowers tumor cells to resist pivotal anti-tumor mechanisms. These include enhanced antigen presentation and cell cycle arrest. Consequently, the tumor evades cytotoxicity mediated by the adaptive immune system. Beyond immune evasion, PTPN2 acts as a direct driver of malignant proliferation and invasion in specific solid tumor subtypes. It achieves this by modulating Src family kinases or the EGFR signaling architecture [3,70]. This complex mechanistic transition from tumor suppression to oncogenesis underscores the context-dependent biological significance of PTPN2 across diverse tumor types and developmental stages. Consequently, this functional duality presents formidable challenges for the development of PTPN2-targeted therapeutics.

In terms of the type and volume of available evidence, current PTPN2-related studies can be broadly grouped into three major categories: human genetic studies, animal model studies, and cellular functional studies. Multiple independent genome-wide association studies (GWAS) have repeatedly confirmed a significant association between PTPN2 and susceptibility to type 1 diabetes across different population cohorts [23,71]. At the animal level, studies using conditional knockout models and disease models have consistently shown that PTPN2 deficiency aggravates immune activation and β-cell injury [2,29]. At the cellular level, a substantial body of mechanistic evidence indicates that PTPN2 participates in inflammatory responses and metabolic regulation primarily through negative regulation of the JAK/STAT signaling pathway [31,56].

Notably, the existing PTPN2 literature is mainly concentrated in the fields of autoimmune diseases and tumor immunology, whereas research on metabolic diseases has emerged relatively later [7]. Among metabolic-related disorders, T1D currently has the strongest evidence base. By contrast, studies on T2D, diabetic complications, MASH, and metabolic bone diseases remain largely dependent on cellular and animal models, with limited large-scale clinical and translational evidence. Overall, the conclusions drawn from different levels of evidence are broadly consistent and support PTPN2 as a regulatory node linking immune regulation with metabolic homeostasis. However, further population-based studies and clinical evidence are needed to clarify its disease-specific functions and translational potential.

## 3. Therapeutic Potential of PTPN2 Targeting in Metabolic Diseases

The mechanistic convergence of PTPN2 signaling networks governing both metabolic homeostasis and tumor immune evasion suggests significant potential for cross-therapeutic applications. Currently, clinical stage PTPN2 inhibitors are predominantly deployed in oncology to reinvigorate anti-tumor immunity against advanced solid malignancies. Nevertheless, these novel small-molecule entities have successfully surmounted historical barriers regarding bioavailability. Consequently, they offer a viable translational strategy for rectifying the metabolic dysregulation associated with Type 2 Diabetes.

Recent breakthroughs involving tumor-targeted agents, notably ABBV-CLS-484 and Compound-182 [72,73], have definitively validated the druggability of PTPN2. Beyond their oncological applications, these findings illuminate novel avenues for therapeutic intervention in metabolic pathologies. Central to the pathological architecture of metabolic disease, PTPN2 governs critical nodes including insulin resistance, β-cell dysfunction, and chronic low-grade inflammation. However, in stark contrast to the rapid clinical advances witnessed in oncology, translational exploration of PTPN2 within the metabolic landscape remains nascent. Moving forward, the strategic leveraging of established oncology pipelines presents a compelling opportunity. Whether achieved through the repurposing of existing inhibitors or the optimization of pharmacophores for metabolic microenvironments, the modulation of PTPN2 represents a promising frontier. Ultimately, this approach holds the potential to deliver novel precision intervention strategies for Type 2 Diabetes.

In the landscape of T2D therapeutics, strategies grounded in high-throughput screening and natural product discovery offer critical avenues for identifying novel PTPN2 inhibitors. Acknowledging that related phosphatases, specifically PTPN1 and PTPN6, are also implicated in insulin resistance [74], multi-target strategies have emerged. Methyl syringate acts as a dual inhibitor of PTPN2 and PTPN6. In mature 3T3-L1 adipocytes, this compound significantly augments glucose uptake and stimulates AMP-activated protein kinase (AMPK) phosphorylation [75]. These effects underscore its potential efficacy against T2D. However, PTPN6 is a key negative regulator in immune cells, and its systemic inhibition may increase the risk of autoimmune adverse effects [76]. Therefore, the in vivo safety and tissue-targeting specificity of this strategy require further investigation. In parallel, research into Momordica charantia (bitter melon) has isolated bioactive components with distinct properties. These constituents demonstrate promising pharmacological potential characterized by a highly selective inhibition of PTPN2, exhibiting an inhibitory efficiency exceeding 70% [77].

Although several PTPN2-targeted therapeutic strategies have shown potential efficacy in experimental models, their clinical translation remains challenging. The catalytic domain of PTPN2 is highly conserved among protein tyrosine phosphatases (PTPs), making it difficult to develop inhibitors with sufficient selectivity. In particular, designing inhibitors that selectively recognize and effectively engage the active pocket of PTPN2, while avoiding binding to structurally homologous family members with important physiological functions, such as PTPN1, remains a major obstacle [73]. To achieve high binding affinity, conventional competitive inhibitors typically incorporate acidic moieties acting as phosphotyrosine (pTyr) mimetics. However, these highly polar groups often compromise the physicochemical properties of the drug candidate. This typically results in poor cell membrane permeability and limited oral bioavailability [72]. Furthermore, the lack of selectivity precipitates off-target interactions which may induce unforeseen cytotoxicity. To circumvent these limitations, the exploitation of allosteric sites represents a promising strategic direction. Validated within the broader PTP superfamily, this approach targets regions distinct from the catalytic center [78,79,80]. Nevertheless, the identification and pharmacological validation of distinct, druggable allosteric pockets capable of conferring high selectivity to PTPN2 remain a frontier requiring extensive investigation.

Beyond the intricacies of molecular mechanisms, a paramount challenge lies in the pleiotropic nature of PTPN2. The phosphatase exerts divergent and occasionally opposing functional roles across distinct tissue compartments. This tissue-specific heterogeneity mandates the engineering of advanced precision delivery systems. Such strategies must be capable of selectively targeting key metabolic organs while strictly sparing the central nervous system and the systemic immune architecture. Furthermore, the chronic systemic inhibition of PTPN2 carries inherent risks. Prolonged suppression may elicit compensatory signaling rewiring or induce adaptive resistance mechanisms. Consequently, these adaptive responses could precipitate unforeseen and deleterious metabolic sequelae.

To circumvent these structural and pharmacokinetic hurdles, researchers have proposed an innovative synergistic modification strategy. This approach employs a dual-lipidation technique applied to the BimBH3 peptide, a PTPN1 inhibitor. Specifically, long-chain fatty acids (C16) and medium-chain fatty acids (C10-C12) were conjugated to the N-terminus and the side chain of the Lys^2^ residue, respectively. Functionally, the long-chain moiety augments metabolic stability and extends the duration of biological activity. Complementing this, the medium-chain component significantly enhances cellular permeability and targeting efficiency. The resulting optimized lipidated analog, designated D6, exhibits potent dual inhibitory activity against both PTPN1 and PTPN2. Furthermore, D6 demonstrates effective transmembrane permeability and successfully restores insulin signaling in HepG2 hepatocytes. In vivo assessments confirm that this analog achieves sustained hypoglycemic efficacy in db/db diabetic mice [81].

A transformative approach to enhancing targeting specificity involves the deployment of Targeted Protein Degradation (TPD) technologies. This strategy utilizes heterobifunctional chimeric molecules, such as Proteolysis Targeting Chimeras (PROTACs) or Lysosome-Targeting Chimeras (LYTACs). Mechanistically, these agents tether PTPN2 to an E3 ubiquitin ligase or a lysosome-targeting receptor, respectively. Consequently, this recruitment precipitates the selective ubiquitin-proteasomal or lysosomal degradation of the target protein. Exemplifying this strategy, the PTPN2-specific PROTAC, TP1L, has successfully demonstrated efficacy in abrogating tumor immune tolerance [82]. Complementing TPD, structure-based drug design offers another avenue for optimizing selectivity. By leveraging in silico computational modeling to scrutinize the subtle microstructural divergences between the active pockets of PTPN2 and its close homolog PTPN1, researchers can identify unique structural features. These insights facilitate the introduction of rational chemical modifications. Ultimately, this enables the engineering of molecules with superior isoform selectivity through precise structural optimization. Representative PTPN2-targeted intervention strategies, their mechanisms of action, and translational challenges are summarized in Table 3.

In conclusion, future investigative endeavors should prioritize dissecting the mechanistic intricacies of PTPN2 within the specific microenvironments characterizing the diabetic state. It is imperative to map the dynamic functional networks and molecular crosstalk of the enzyme across distinct disease stages and key metabolic organs, including pancreatic islets, adipose tissue, and the liver. Such comprehensive mapping is essential for circumventing current bottlenecks in drug discovery. Concurrently, the development of intelligent, tissue-tropic delivery systems, coupled with the integration of therapeutic strategies into robust translational frameworks, is critical. These synergistic efforts will successfully facilitate the conversion of fundamental PTPN2 biology into clinically viable precision therapies. Ultimately, this translational trajectory aspires to deliver transformative treatment paradigms, offering renewed hope for the global diabetic population.

## 4. Outlook and Future Perspectives

Collectively, current evidence indicates that PTPN2 exerts predominantly protective effects in diabetes and its complications. For example, in pancreatic β-cells, the liver, and the kidney, PTPN2 appears to confer beneficial effects by suppressing inflammatory signaling, maintaining cellular homeostasis, and attenuating tissue injury. However, several recent studies have reported findings that are not fully consistent with this protective model. In hypothalamic AgRP neurons, PTPN2 can inhibit insulin signaling through dephosphorylation of the insulin receptor, thereby promoting central insulin resistance. Similarly, in skeletal muscle cells exposed to inflammatory stimuli, PTPN2 upregulation has been associated with reduced insulin sensitivity. In addition, in the field of cancer research, PTPN2 inhibition has been shown to enhance IFN-γ signaling and T cell-mediated antitumor immune responses. These observations suggest that the biological functions of PTPN2 cannot be simply categorized as either disease-promoting or disease-protective, but are instead strongly shaped by tissue context and the surrounding microenvironment.

This functional duality may be attributable to the broad substrate spectrum of PTPN2 and the complexity of the signaling networks it regulates. As a non-receptor protein tyrosine phosphatase, PTPN2 can negatively regulate inflammation-related pathways, such as JAK/STAT signaling, while also directly acting on the insulin receptor and other receptor tyrosine kinase-associated signaling pathways. Therefore, its ultimate biological effects largely depend on the dominant substrates and signaling pathways present in specific tissues. When inflammation is the primary driver of disease initiation and progression, PTPN2 may exert protective effects by limiting excessive inflammatory responses. In contrast, when insulin signaling is the predominant regulatory pathway, PTPN2-mediated dephosphorylation may weaken insulin signal transduction and thereby produce unfavorable metabolic effects. These observations suggest that PTPN2 is better viewed as a dynamic regulator of signaling intensity rather than as a unidirectional protective or pathogenic factor. Overall, current evidence supports the concept that PTPN2 functions as an important node linking immune regulation and metabolic homeostasis. Its functional duality not only helps explain the divergent findings observed across different disease contexts but also suggests that future PTPN2-targeted therapeutic strategies may need to be tailored according to tissue type and disease stage, rather than relying on global activation or systemic inhibition.

It should be noted that, beyond acting as a negative regulator of downstream signaling pathways, PTPN2 expression and activity are themselves dynamically regulated by several upstream factors. Among these, oxidative stress has emerged as an important regulatory input. As a member of the classical PTP family, PTPN2 contains a conserved catalytic cysteine residue that is highly sensitive to the cellular redox state. Reactive oxygen species (ROS) generated under hyperglycemic or lipotoxic conditions can modulate its phosphatase activity through reversible oxidation [83], thereby altering its ability to regulate signaling pathways such as JAK/STAT. In addition, TNF-α is not only a signaling target regulated by PTPN2 but can also induce PTPN2 expression, suggesting that PTPN2 may participate in a negative feedback loop during TNF-α-mediated inflammatory responses [84]. Together, these findings indicate that PTPN2 is not a unidirectional signaling molecule, but rather a regulatory node at the interface between oxidative stress and inflammatory signaling, contributing to the maintenance of cellular signaling homeostasis.

Notably, most current studies on PTPN2 function are still derived from cellular experiments and genetically modified animal models, whereas PTPN2 variants associated with human diseases more commonly affect gene expression rather than cause complete loss of protein function. Therefore, phenotypic differences observed across studies may reflect genuine tissue-specific effects, but they may also be influenced by experimental models, disease stage, and local microenvironmental conditions. Future studies integrating population genetics, single-cell omics, and spatial transcriptomics will be needed to further define the dynamic regulatory mechanisms of PTPN2 across different cell types and disease stages.

In addition, most existing studies on PTPN2 have not systematically examined sex-dependent differences. However, emerging evidence from immunometabolic research suggests that sex may influence JAK/STAT signaling regulation and autoimmune susceptibility through differences in hormone levels, X chromosome-linked immune gene expression, and the magnitude of inflammatory responses [85,86]. Therefore, sex-specific differences in PTPN2 expression and function warrant further systematic investigation and may represent a potential factor contributing to interindividual variability in autoimmune diseases.

Although PTPN2 has long been considered to exert protective effects in maintaining metabolic homeostasis, recent advances in pharmacological inhibitors and targeted degradation technologies suggest that it may also represent an actionable therapeutic target in specific tissues and pathological contexts. Small-molecule or peptide-based inhibitors, represented by methyl syringate and the dual fatty acid-conjugated peptide D6, have shown potential to improve insulin sensitivity and glucose metabolism in preclinical studies. Meanwhile, emerging technologies such as PROTACs provide new opportunities for the selective modulation of PTPN2. These findings further suggest that the therapeutic value of PTPN2 may not depend on simple activation or inhibition, but rather on precise, context-dependent intervention tailored to tissue type and disease stage.

In summary, PTPN2 functions as a critical nexus bridging metabolic homeostasis and immune regulation, offering a novel therapeutic dimension for abrogating metabolic inflammation. Concurrently, the synergistic integration of structural biology, Artificial Intelligence-driven Drug Discovery (AIDD), and clinical medicine holds immense promise for solidifying the therapeutic utility of PTPN2 in complex metabolic disorders. Ultimately, this multidisciplinary approach may help refine future PTPN2-targeted strategies aimed at improving metabolic regulation while reducing the risk of unwanted immune-related effects.

## 5. Conclusions

PTPN2 (also designated as TC-PTP) stands as a paramount regulator within the non-receptor protein tyrosine phosphatase superfamily. It is indispensable for the maintenance of immune tolerance, the preservation of insulin sensitivity, the protection of β-cell functional integrity, and the orchestration of systemic metabolic homeostasis. Mechanistically, PTPN2 exerts these effects by executing the precise dephosphorylation of pivotal signaling nodes within the JAK/STAT and Insulin Receptor pathways, as well as those governing cellular apoptosis. This review has systematically synthesized the multifaceted molecular landscapes of PTPN2 across a broad spectrum of metabolic pathologies, encompassing Type 1 and Type 2 Diabetes, associated diabetic complications, metabolic dysfunction-associated steatohepatitis (MASH), and metabolic bone disorders. Special emphasis has been placed on the critical analysis of emerging targeted therapeutic strategies for Type 2 Diabetes. Ultimately, this synthesis provides novel insights and strategic perspectives intended to accelerate the clinical translation of PTPN2-centric interventions.

## Figures and Tables

**Table 1:** Domain Architecture and Subcellular Localization of PTPN2 Isoforms.

**PTPN2 Isoform**	**Domain Architecture**	**Subcellular Localization**
48 kDa Isoform (PTPN2-TC48)	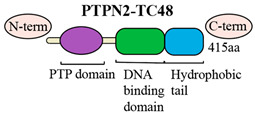	Endoplasmic Reticulum (ER) Retention: Characterized by a C-terminal hydrophobic tail that masks the bipartite nuclear localization signal (NLS). This structural feature anchors the protein to the ER membrane, restricting it to the reticular network [17].
45 kDa Isoform (PTPN2-TC45)	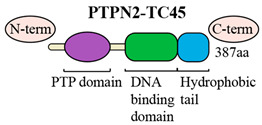	Nuclear Localization & Cytoplasmic Shuttling: Lacks the hydrophobic tail, exposing the C-terminal NLS which drives predominant nuclear entry. Under specific cellular stress conditions (e.g., EGFR activation), it passively translocates into the cytoplasm to execute substrate dephosphorylation [7].

**Table 2:** PTPN2-Mediated Osteoimmunological Regulation of Bone Homeostasis.

Cell Type	Osteoimmunological Impact	Molecular Mechanism
Macrophages	Inhibits activation and bone resorption: Suppresses osteoclast precursor differentiation and limits M1 macrophage polarization and pro-inflammatory cytokine production.	Polarization Regulation: Limits macrophage responsiveness to LPS and IFN-γ to maintain M1/M2 balance and suppress M1-driven bone resorption [60].
		CSF-1/CSF-1R Axis: Dephosphorylates CSF-1R (Y807) and ERK to suppress CSF-1-induced osteoclast precursor differentiation [56,61].
		Inflammasome Modulation: Dephosphorylates JNK to block NLRP3 inflammasome assembly and IL-1β release, thereby attenuating inflammatory bone resorption [62].
T cells	Preserves skeletal homeostasis: Stabilizes Treg function and restricts pathogenic Th1/Th17 expansion.	Foxp3 Stability: Dephosphorylates STAT3 to prevent IL-6-driven Foxp3 loss, maintaining the suppressive Treg phenotype [63].
		Differentiation Kinetics: Dephosphorylates JAK1, JAK3, and STAT5 to negatively regulate IL-2 signaling and tune Treg expansion [64].
		TCR Activation Control: Inhibits TCR signaling and Lck hyperphosphorylation to suppress inflammatory responses [29].
B cells	Regulates lymphopoiesis and OPG/RANKL balance: Ensures normal B cell development and prevents pathological osteoclastogenesis.	Pro-Maturation Support: Inhibits IFN-γ/STAT1 signaling to promote maturation from pre-B to immature B cells [65].
		Anti-Proliferative Control: Suppresses IL-21/STAT3 signaling to limit excessive B cell proliferation [66].

**Table 3:** Representative PTPN2-targeted intervention strategies, mechanisms of action, and translational challenges.

Inhibitor/Intervention Strategy	Target and Molecular Mechanism	Selectivity and Structural Basis	Pharmacokinetics and In Vivo Distribution	Major Clinical limitations and Safety Concerns
ABBV-CLS-484 [72]	Orthosteric dual PTPN1/PTPN2 inhibitor	High-affinity inhibitor targeting the highly conserved catalytic pocket	Favorable in vivo exposure profile; enables systemic PTPN1/PTPN2 inhibition	Potential risk of systemic autoimmunity; long-term safety and suitability for chronic metabolic indications remain to be established
D6 (lipidated peptide) [81]	Dual PTPN1/PTPN2 lipidated peptide inhibitor	Conjugation with medium- and long-chain fatty acids enhances cellular permeability	Prolonged in vivo half-life; shows clear liver enrichment tendency in animal models	Potential peptide stability and long-term safety concerns require further validation
TP1L (PROTAC) [82]	Selective PTPN2 degrader based on E3 ligase recruitment	Achieves high functional selectivity through targeted protein degradation	Large PROTAC molecular size; cellular permeability and in vivo distribution require further optimization	Large molecular weight; drug-like properties and in vivo delivery require further optimization

## Data Availability

No new data were created or analyzed in this study. Data sharing is not applicable to this article.

## Abbreviations

The following abbreviations are used in this manuscript:

PTPs	Protein tyrosine phosphatases
DUSPs	Dual-specificity phosphatases
PTPN2	Protein tyrosine phosphatase non-receptor type 2
TC-PTP	T-cell protein tyrosine phosphatase
PTPN1	Protein tyrosine phosphatase non-receptor type 1
NLS	Nuclear localization signal
T1D	Type 1 diabetes
SNPs	Single nucleotide polymorphisms
TCR	T-cell receptor
JAK	Janus kinase
STAT	Signal transducer and activator of transcription
CVB	Coxsackievirus B
ISGs	Interferon-stimulated genes
AgRP	Agouti-related peptide
IR	Insulin receptor
BAT	Brown adipose tissue
HGP	Hepatic glucose production
HFD	High-fat diet
IRS1	Insulin Receptor Substrate 1
IFN-γ	Interferon-γ
TNF	Tumor necrosis factor
IL	Interleukin
MASH	Metabolic dysfunction-associated steatohepatitis
MASLD	Metabolic dysfunction-associated steatotic liver disease
HCC	Hepatocellular carcinoma
CSF-1R	Colony-stimulating factor 1 receptor
Treg	Regulatory T cell
PROTACs	Proteolysis Targeting Chimeras
